# Crimean-Congo Hemorrhagic Fever Virus for Clinicians—Diagnosis, Clinical Management, and Therapeutics

**DOI:** 10.3201/eid3005.231648

**Published:** 2024-05

**Authors:** Maria G. Frank, Gretchen Weaver, Vanessa Raabe, State of the Clinical Science Working Group of the National Emerging Pathogens Training, Education Center’s Special Pathogens Research Network

**Affiliations:** Denver Health and Hospital Authority, Denver, Colorado, USA (M.G. Frank);; University of Colorado School of Medicine, Denver (M.G. Frank);; University of Massachusetts Chan Medical School, Worchester, Massachusetts, USA (G. Weaver);; New York University Grossman School of Medicine, New York, New York, USA (V. Raabe)

**Keywords:** Crimean-Congo hemorrhagic fever, viruses, zoonoses, CCHFV, bunyaviruses, viral hemorrhagic fever, countermeasures, vaccine, treatment

## Abstract

Crimean-Congo hemorrhagic fever virus (CCHFV) is the most geographically widespread tickborne viral infection worldwide and has a fatality rate of up to 62%. Despite its widespread range and high fatality rate, no vaccines or treatments are currently approved by regulatory agencies in the United States or Europe. Supportive treatment remains the standard of care, but the use of antiviral medications developed for other viral infections have been considered. We reviewed published literature to summarize the main aspects of CCHFV infection in humans. We provide an overview of diagnostic testing and management and medical countermeasures, including investigational vaccines and limited therapeutics. CCHFV continues to pose a public health threat because of its wide geographic distribution, potential to spread to new regions, propensity for genetic variability, potential for severe and fatal illness, and limited medical countermeasures for prophylaxis and treatment. Clinicians should become familiar with available diagnostic and management tools for CCHFV infections in humans.

Approximately 10,000–15,000 cases of human Crimean-Congo hemorrhagic fever (CCHF) occur annually worldwide (*1*–*3*). However, more definitive case numbers are difficult to ascertain because up to 88% of infections are thought to be subclinical (*1*–*3*), unrecognized, or occurring in locations with limited disease surveillance or laboratory testing capability (*4*,*5*). CCHF virus (CCHFV) causes a spectrum of human clinical manifestations, ranging from asymptomatic infection to a severe hemorrhagic fever marked by shock and multiorgan failure (Figure). During CCHF outbreaks, the case-fatality rate ranges from 5%­ to 30% (*1*), and some published case series have reported fatality rates up to 62% (*6*). Disease caused by CCHFV infection is limited to humans, but asymptomatic transient viremia lasting up to 15 days has been documented in livestock and wild animals (*7*). Severe or fatal disease correlates with an exuberant proinflammatory immune response leading to vascular dysfunction, disseminated intravascular coagulation, multiorgan failure, and shock (*8*). Although the detection of IgM (usually present as early as day 4–5 of illness) and IgG (usually detectable after days 7–9 of illness) correlates with declining viremia, fatal cases of CCHF often mount no or very late immune responses (*9*). However, the antibody response to CCHFV does not correlate with disease outcome or protection from vaccines. That feature of CCHF, combined with a paucity of available animal models (*10*), makes vaccine and treatment research challenging. The US Food and Drug Administration (FDA) has not approved any vaccines or treatments for CCHF. Ribavirin is commonly used for treatment but clinical evidence regarding its benefit is mixed. Another antiviral medication, favipiravir, shows promise in animal models but has rarely been used in human CCHF management. Vaccine candidates are mostly in preclinical development, and few have advanced to human clinical trials to date.

**Figure:**
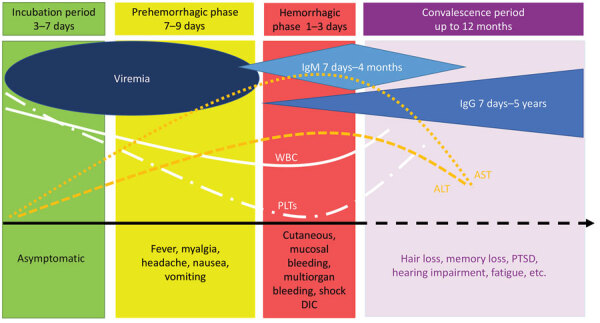
Overview of Crimean-Congo hemorrhagic fever virus symptom onset, clinical course, and diagnostic testing timeframes. ALT, alanine aminotransferase; AST, aspartate aminotransferase; DIC disseminated intravascular coagulation; PLTs, platelets; PTSD, post-traumatic stress disorder; WBCs, white blood cells.

This third article in a 3-part series summarizing the main aspects of CCHF is intended to provide clinicians with an overview of diagnostic testing, management, and medical countermeasures for CCHF. The first article focuses on the virology, pathogenesis, and pathology of CCHF (*11*) and the second on epidemiology, clinical features, and prevention and control of CCHF (*12*). 

## Methods

For this paper, we conducted a focused review of National Center for Biotechnology’s MeSH (Medical Subject Headings, https://www.ncbi.nlm.nih.gov/mesh) and PubMed (https://pubmed.ncbi.nlm.nih.gov) search strings customized for CCHF and CCHFV. We focused our review on the past 10 years and on human data, when available. We included older relevant data and animal data where appropriate. We conducted title, abstract, and full text reviews of relevant manuscripts, reviews, and book chapters. We also completed bibliography scans on review articles and meta-analyses.

### Diagnostic Testing

The nonspecific CCHF characteristics make a high index of suspicion, on the basis of epidemiologic history, clinical signs and symptoms, and initial laboratory findings, key for early diagnosis and initiation of aggressive treatment. Delays in diagnosis and hospitalization are common and have occurred in up to 68% of patients in Turkey and led to increased mortality rates when compared with patients whose infections are diagnosed early (*13*).

Although many laboratory assays have been developed for diagnosing CCHF, the availability and Biosafety Level (BSL) requirements for safe specimen handling vary widely between countries. Sweden, Switzerland, France, Germany, Italy, and the United Kingdom recommend BSL-4 for CCHFV diagnostic assays (*14*). Conversely, the United States, South Africa, Kazakhstan, Slovenia, and Georgia allow diagnostic tests to be performed in BSL-3 laboratories, and Bulgaria, Turkey, and Serbia recommend BSL-2 laboratories (*15*). For most testing modalities, other than viral culture, viral inactivation of the sample can be performed to downgrade the BSL requirement (*16*,*17*).

Diagnosis can be obtained either by viral detection or identification of an immune response against CCHFV (Table), and test selection is guided by clinical phase. Although most viral detection tests, either viral culture, nucleic acid amplification tests, or viral antigen detection assays, will have greater sensitivity than immunoassays for diagnosis during the prehemorrhagic or early hemorrhagic phases, serologic testing is reserved for a delayed diagnosis or beyond day 5 after symptom onset (*17*) (Table; Figure). If a negative antibody test is obtained during the second week of illness in a patient suspected to have CCHF, a direct viral assay might be warranted for diagnostic clarification (*17*). Undetectable IgM and IgG in CCHF patients with fatal outcomes have been described (*9*). Whether those patients succumbed to CCHF because of failure to mount an antibody response, a rapidly progressive clinical course with fatal outcome before day 7, or formation of immune complexes that made antibodies undetectable is unclear (*9*).

**Table:** Advantages and disadvantages of various diagnostic tests for CCHFV*

Test selection	Timing	Advantages	Disadvantages
Viral detection†			
Viral culture‡	Early after symptom onset	Detects a wide diversity of CCHFV strains	Requires BSL-3 or BSL-4 laboratory, which are not readily available in endemic areas. Requires several days to yield a result.
NAAT, RT-PCR	<10–12 days after symptom onset	If samples are inactivated, then NAAT can be run in BSL-2 or BSL-3 facilities. Several multiplex assays available, and some can quantify viral load.	Variable sensitivity depending on match between primers and infecting strain. Sensitivity and specificity can vary by geographic region. Better sensitivity (80%) when PCR combinations used, e.g., rRT-PCR and conventional PCR or rRT-PCR and nested PCR (*17*).
Viral IgG detection			
ELISA	<5­–9 days after symptom onset	Timely results. Viral inactivation can be performed. Requires less laboratory specialization.	Decreased sensitivity after CCHFV antibodies are detectable.
Immunohistochemistry	<5­–9 days after symptom onset	Can assist in retrospective diagnosis for fatal cases.	Requires biopsy or necropsy samples.
Immune response, serology			
IgM ELISA§ or IFA¶	Detectable 7–9 days after symptom onset; peak 2–3 weeks; declines to low levels by month 4	ELISA sensitivity 87.8%, specificity 98.9. IFA sensitivity 93.9%, specificity 100% (*17*).	Commercially available kits for research but not for clinical laboratories; variable geographic sensitivity; IgM might not be detectable in fatal cases
IgG ELISA§, IFA¶, or Luminex xMAP	Detectable 1–2 d after IgM, peaks 2 wks–5 mo; detectable for <3 y	ELISA sensitivity 80.4%, specificity 100%. IFA sensitivity 86.1% specificity 100% (*17*).	Commercial ELISA and IFA kits available for research but not for clinical laboratories; variable geographic sensitivity; IgM might not be detectable in fatal cases
Neutralizing antibodies#	>10 days after illness onset	Can be performed in BSL-2 facilities	Takes several days to perform. Not routinely used for diagnostic purposes.

*BSL, Biosafety Level; CCHFV, Crimean-Congo hemorrhagic fever virus; RT-PCR, reverse transcription PCR; rRT-PCR, real-time RT-PCR; Ddx, differential diagnoses; IFA: immunofluorescence assays; Nabs, neutralizing antibodies. †Recommended when patient is viremic. Could be performed as cell culture or intracerebral inoculation of mice. ‡RT-PCR could be real-time, conventional, nested or a combination §VectroCrimea-CHF (Vector-Best, https://en.vector-best.ru). ¶Crimean Congo Fever Mosaic 2 (Euroimmun, https://www.euroimmun.com). #Pseudoplaque or plaque reduction neutralization tests for CCHFV viral-like particles.

Direct viral detection tests are useful during viremic stages of CCHF. Viral cultures using cell lines or intracerebral inoculation of suckling mice can detect a wide diversity of CCHFV strains; however, viral cultures are time-consuming, and results can take several days. Another challenge is the paucity of BSL-3 and BSL-4 laboratories able to safely perform viral cultures in endemic areas (*17*). Nucleic acid amplification tests, such as reverse transcription PCR (RT-PCR), can be useful for diagnosis until up to days 10−12 of illness. Assays can be run in inactivated samples in BSL-2 and BSL-3 facilities, but correct inactivation methods need to be selected for compatibility with the chosen diagnostic test (*15*,*16*). Some of those assays are designed as multiplex assays instead of CCHFV-specific and provide breadth to rule out other viral hemorrhagic fevers (*17*). Assays capable to detecting viral load also are available (*17*). However, accuracy of those tests varies and is affected by the match between the PCR primers used and the viral strain because of the wide genetic diversity of CCHFV (*17*). Accuracy can be improved by using real-time RT-PCR (rRT-PCR) or a combination of rRT-PCR and conventional RT-PCR or rRT-PCR and nested RT-PCR (*17*). Combining assays can increase accuracy to 80% from 66% of reference samples when using conventional RT-PCR alone and from 46% when using nested RT-PCR alone (*17*). 

Viral antigen detection tests, such as ELISA for serum or immunohistochemistry for tissue from biopsies or autopsy samples, also can be used early in the disease. Those assays require a lower level of laboratory sophistication, can be done on inactivated samples, and offer timely results; however, assay sensitivity decreases as antibodies become detectable. IgM can be detectable as early as 4–5 days and usually by 7–9 days after symptom onset, peaks within 2–3 weeks, and declines to an almost undetectable level by month 4 from symptom onset (*9*,*16*,*17*). IgG typically becomes detectable 1–2 days after IgM (usually 7–9 days after illness onset), peaking in some patients between weeks 2–3 and between 2–5 months in some other patients. IgG remains detectable for at least 3 years (*9*,*16*,*17*). Neutralizing antibodies, although not routinely tested in the clinical setting, often can be detected by day 10 with variable titers (*9*). 

CCHF diagnosis can be confirmed not only by the direct viral identification methods described, but also by evidence of a serologic response consistent with acute infection. CCHFV serologic testing is typically recommended 5–7 days after symptom onset; ELISA and immunofluorescence assays are the most common (*16*) (Figure). ELISA results are considered consistent with acute infection if either CCHFV IgM is detected or a 4-fold increase in CCHFV IgG titers occurs between serial blood samples. Some CCHFV antibody assays are known to cross-react with Nairobi sheep disease serogroup, which also causes human disease in some CCHF endemic areas, and with Hazara virus, a member of the CCHFV group with no documented human disease (*18*). Several ELISA kits are commercially available for research but not for clinical diagnostic testing, and their sensitivity and specificity are also susceptible to CCHFV genetic variability (*16*). However, some of those assays have sensitivities >80% and specificities close to 100% (*17*). One commercially available immunofluorescence assay demonstrated a sensitivity of 93.9% for IgM and 86% for IgG and 100% specificity for both (*17*) (Table). No commercial rapid CCHF diagnostic tests are available for clinical use (*16*,*17*,*19*).

In the United States, testing of clinical specimens for CCHFV at designated reference laboratories should be arranged by consulting with respective local public health authorities and coordinating with the Centers for Disease Control and Prevention. CCHFV is a notifiable communicable disease in the United States and Europe and is considered a category A priority pathogen by the National Institute of Allergy and Infectious Diseases (NIAID; https://www.niaid.nih.gov/research/emerging-infectious-diseases-pathogens). Thus, clinicians should coordinate with public health authorities for collecting and shipping clinical samples and should follow requirements by the US Department of Transportation Hazardous Materials Regulations, 49 CFR 171–180 (https://www.ecfr.gov/current/title-49/subtitle-B/chapter-I/subchapter-C), and the International Air Transport Association Dangerous Goods Regulations (https://www.iata.org/en/publications/dgr).

### Medical Countermeasures

No FDA-approved medications or vaccines are available to prevent or treat CCHF, nor are any approved by the European Medicines Agency. In this section, we describe evidence for off-label use of existing medications and upcoming investigational countermeasures that are in development and have been assessed for prophylactic or therapeutic effects on CCHFV infection in humans or animal models. The first article in this series should serve as a reference on virologic features when reviewing medical countermeasures and vaccine sections (*11*).

#### Preexposure Prophylaxis and CCHF Vaccines 

A single vaccine is available to prevent CCHF in humans, produced by BulBio-NCIPD Limited (https://wwww.bulbio.com), but its licensure is limited to Bulgaria. This vaccine originally was developed in the former Soviet Union in 1970 and has been used in at-risk populations in Bulgaria, primarily military and medical personnel, since 1974 (*20*). The vaccine consists of chloroform and heat inactivated CCHFV strain V42/81 isolated from suckling mouse brain tissue (*20*). The vaccine series is administered in 2 intramuscular doses 30 days apart, a 3rd dose at 1 year, and subsequent booster doses every 5 years (*20*). The vaccine has been shown to elicit CCHFV IgG but with low viral neutralization activity and T-cell responses to the CCHFV nucleoprotein (*21*). No vaccine effectiveness data are available. A 4-fold decrease in CCHFV diagnoses in Bulgaria was demonstrated in the 21 years after introduction of that vaccine; however, the degree to which the decrease is attributable to vaccination versus other measures remains unclear (*20*).

Aside from the inactivated vaccine available in Bulgaria, only 1 vaccine candidate, an inactivated vaccine derived from cell culture, has advanced to human clinical trials (https://www.ClinicalTrials.gov, no. NCT03020771). Although a phase 1 trial has been completed, no results were available by early 2023. Several other vaccine candidates are in preclinical development. Those vaccines primarily target the CCHFV glycoprotein, nucleoprotein, or both, including DNA-based (*22*–*29*), RNA-based (*30*,*31*), protein subunit–based (*32*–*34*), viral replicon particle­–based platforms (*35*–*38*), and recombinant viral vector–based platforms that use bovine herpesvirus type 4, human adenovirus 5, modified vaccinia Ankara, and vesicular stomatitis virus (*39*–*44*). Vaccine-induced protection might be elicited by vaccines containing either the CCHFV glycoprotein or nucleoprotein in preclinical studies, but not consistently across vaccine platforms expressing the same antigen (Appendix 1 Table).

The World Health Organization has identified development of CCHF vaccines as a priority (*45*) but faces multiple challenges, including the high degree of genetic diversity between CCHFV strains, the need for high biocontainment laboratories to perform challenge studies, and the limited animal models in which CCHF disease can be replicated. Animal models amenable to vaccine studies were not available until lethal CCHF disease models in mice with deficits in type 1 interferon signaling pathways (STAT1−/− and IFNAR−/− mice) were identified in 2010 (*46*,*47*). Recently developed models of CCHF disease in humanized mice, in cynomolgus macaques (*Macaca fascicularis*), and among immunocompetent mice using a mouse-adapted CCHFV variant provide additional options for future CCHF vaccine studies (*48*–*50*). The lack of immune correlates of protection against CCHFV additionally poses a challenge for vaccine development. Neither CCHFV antibody titers nor neutralizing antibody titers correlate with vaccine-induced protection against disease or survival in animal models (*9*,*10*,*25*,*26*,*32*,*43*). However, recent data from a novel repRNA vaccine expressing CCHFV nucleoprotein suggest that a single dose of this vaccine could induce robust and protective immunity in mice (*30*). Some studies suggest that both humoral and cellular immune responses might be required for full protection against CCHF disease and death (*30*,*42*). 

#### Postexposure Prophylaxis

Ribavirin (1-β-D-ribofuranosyl-1,2,4-triazole-3-carboxamide) is a purine nucleoside analog that acts against a wide range of viruses (Appendix references *51*,*52*). Ribavirin has multiple potential mechanisms of antiviral activity and in vitro antiviral activity against CCHFV (Appendix 2 references *51*,*52*). The effectiveness of oral ribavirin prophylaxis for preventing CCHF is unknown, but it has been used as postexposure prophylaxis among healthcare workers with known CCHFV exposures (Appendix 2 references 53–56). The optimal dosing and duration of postexposure prophylaxis is unclear; postexposure ribavirin regimens reported to date for CCHFV include total doses ranging from 1,200 to 4,000 mg daily and doses administered from 2 to 4 times daily for 5–14 days, with or without a loading dose (*21*) (Appendix 2 references *53–57*). CCHFV seroconversion has been reported among healthcare workers who were administered postexposure ribavirin prophylaxis after sustaining breaches in personal protective equipment while managing CCHF patients (Appendix 2 references *54,58*). Mild symptoms in those healthcare workers initially were attributed to side effects from ribavirin (Appendix 2 references *54,58*). Ribavirin frequently causes side effects, including fatigue, gastrointestinal symptoms, headache, hemolytic anemia, and laboratory abnormalities (Appendix 2 references *54,55*). Ribavirin is contraindicated during pregnancy and has an FDA category X rating because of potential embryotoxic and teratogenic effects (Appendix 2 reference *59*).

#### Antiviral Drug Treatments

##### Ribavirin

In addition to postexposure prophylaxis, ribavirin has been used to treat CCHF. Ribavirin reduces CCHFV viral loads in murine models (Appendix 2 references *60,61*). However, similar viremia reductions have not been observed among infected humans treated with ribavirin compared with untreated control patients (Appendix 2 references *62,63*).

Evidence from human studies of ribavirin for CCHF treatment mainly consists of case series, case-control studies that use historical controls, and retrospective analyses, but few randomized clinical trials have been conducted, and meta-analyses identified potential for bias in multiple studies (Appendix 2 references *64–67*). In addition to variable study design, comparison of ribavirin effectiveness across studies is challenging because study outcomes could be influenced by other heterogeneous factors, such as differences in the administration route (oral vs. intravenous) and dosing of ribavirin, timing of ribavirin initiation, co-administration of other potential disease-modifying medications, severity of patients analyzed, and variation in predominant CCHFV strains in different geographic regions. 

A prospective, randomized clinical trial of oral ribavirin for CCHF treatment conducted in Turkey compared ribavirin with supportive therapy alone (Appendix 2 reference *68*). In that study, patients were administered 30 mg/kg ribavirin as a loading dose, then 15 mg/kg every 6 hours for 4 days, after which they received 7.5 mg/kg every 8 hours for 6 days. The researchers observed no substantial differences in death, hospitalization duration, time to normalization of transaminases, or percentage of patients requiring platelet transfusions (Appendix 2 reference *68*). Several meta-analyses reveal mixed results for effects of ribavirin on CCHF, ranging from no clear survival benefit to a 1.7-fold reduction in mortality rates among CCHF patients treated with ribavirin compared with those not receiving ribavirin (Appendix 2 reference *64–67*).

##### Favipiravir 

Favipiravir (6-fluoro-3-hydroxy-2-pyrazinecarboxamide) is a pyrazine analog that inhibits RNA polymerase activity in a wide variety of viruses. In vitro studies suggest that premature chain termination induced by favipiravir exceeds that of ribavirin for CCHFV and demonstrate synergistic antiviral effects when ribavirin and favipiravir are combined (Appendix 2 references *69,70*). 

Favipiravir is not licensed for use in the United States but is licensed for treatment of novel influenza A in Japan. Early favipiravir treatment for CCHFV infection reduces viral loads and clinical signs in both murine and macaque CCHF models, but prolonged viral detection and occasional late-onset CCHF disease were observed in mice (Appendix 2 references *60,71*). In lethal CCHFV challenge mouse models, treatment with favipiravir enhanced disease survival, even when initiated as late as 6 days postinfection (Appendix 2 references *60,70*). Only 1 case of human CCHF treatment with favipiravir has been described: a patient hospitalized with CCHFV and SARS-CoV-2 co-infection was treated with 1,600 mg favipiravir twice daily on day 1, then 600 mg twice daily for 4 days; the patient subsequently recovered (Appendix 2 reference *72*). Favipiravir induces teratogenicity in animal models and should be avoided in pregnant and lactating women, if possible. Additional adverse effects include the potential for gastrointestinal distress (e.g., nausea, vomiting, or diarrhea), increased bilirubin levels, transaminitis, QTc prolongation, and hyperuricemia (Appendix 2 reference *73*).

#### Other Treatments

Supportive therapy remains the mainstay of CCHF treatment. Such therapy includes fluid replacement, management of electrolyte disturbances, blood product replacement for critically low levels and coagulopathy (e.g., fresh frozen plasma, packed red blood cells, or platelets), treatment of secondary infections, and external support for organ dysfunction (e.g., hemodialysis, mechanical ventilation) when necessary (Appendix 2 references *74–77*). Aspirin and nonsteroidal antiinflammatory drugs should be avoided because of the potential inhibition of platelet aggregation or agglutination.

Therapeutic plasma exchange and plasmapheresis have been used to treat CCHF, but the clinical benefits of those measures remain unclear because data are limited to individual case reports or small case series (Appendix 2 references *78–81*). In patients with CCHF-related hemophagocytic lymphohistiocytosis, use of intravenous immunoglobulin or high dose steroids, in addition to blood product transfusions, has been reported (Appendix 2 references *82,83*). In a study of 35 CCHF patients in Iran, high dose methylprednisolone (10 mg/kg for 3 days, followed by 5 mg/kg for 2 days) administered with ribavirin to patients with platelet counts <50,000/mL resulted in higher platelet and leukocyte counts and decreased need for transfusions compared with ribavirin alone, but no difference in deaths was observed (Appendix 2 reference *84*). In 1 retrospective study, fewer deaths were observed among patients with severe CCHF who received both corticosteroids and ribavirin therapy compared with those treated with ribavirin alone, but no statistically significant decrease in deaths was observed among patients with mild or moderate CCHF treated with this combination (Appendix 2 reference *85*). In 1 meta-analysis, an additional decrease in deaths was observed for corticosteroids in addition to ribavirin compared with ribavirin alone (Appendix 2 reference *65*).

Hyperimmune immunotherapy for CCHF using pooled serum or plasma harvested from CCHF survivors or CCHF vaccine recipients has been reported, but its effectiveness is unknown because its use has only been reported in small case series (Appendix 2 references *86–88*). Hyperimmune serum for CCHF treatment is not approved by either the FDA or the European Medicines Agency. Monoclonal antibodies for CCHF are in preclinical development and show improved survival among mouse models, but the degree of protection conferred by some monoclonal antibodies varied depending on the infecting CCHFV strain (Appendix 2 references *89,90*).

## Conclusion

CCHFV poses a continued public health threat given its wide geographic distribution, potential to spread to new regions, propensity for genetic variability, and potential for severe and fatal illness. Although infection control measures can be effective in reducing the risk for CCHFV transmission within community and healthcare settings, those measures require correct and consistent application. An urgent need exists for new CCHF diagnostic tests, prophylaxes, and treatments. The current lack of licensed effective therapeutic and prophylactic drugs, gaps in our understanding of CCHFV pathogenesis and immunology, and slow progression in development of CCHF medical countermeasures are in part related to the dearth of animal models and high level of biosafety precautions needed to safely work with CCHFV.

In conclusion, to promptly diagnose CCHF, clinicians should have a high index of suspicion, collect a comprehensive travel and epidemiologic history, and perform a thorough clinical evaluation. Evidence to demonstrate human benefit from off-label use of ribavirin and favipiravir is disparate. Because few medical countermeasures are available for prophylaxis and treatment, supportive care remains the treatment standard for CCHF disease management. New CCHF diagnostic tests, prophylaxis, and treatments are urgently needed. Given its wide range and potential for severe outcomes, clinicians should become familiar with available diagnostic and management tools for CCHFV infections in humans. 

Appendix 1 Additional information for clinicians on Crimean-Congo hemorrhagic fever virus diagnosis, clinical management, and therapeutics.

Appendix 2 Additional references for clinicians on Crimean-Congo hemorrhagic fever virus diagnosis, clinical management, and therapeutics.
