# Supplementary material for: Crimean-Congo Hemorrhagic Fever Virus for Clinicians—Diagnosis, Clinical Management, and Therapeutics
Source: Emerg Infect Dis. 2024 May;30(5):864–73. doi: 10.3201/eid3005.231648 (PMC11060459; doi:10.3201/eid3005.231648)
Supplement: Appendix 1 — Additional information for clinicians on Crimean-Congo hemorrhagic fever virus diagnosis, clinical management, and therapeutics. [file 23-1648-Techapp-s1.pdf]

Article DOI: https://doi.org/10.3201/eid3005.231648

EID cannot ensure accessibility for supplementary materials supplied by authors. Readers who have difficulty accessing supplementary content should contact the authors for assistance.

Crimean-Congo Hemorrhagic Fever Virus for Clinicians—Diagnosis, Clinical Management, and Therapeutics

Appendix 1

Appendix 1 Table. CCHF Vaccine Candidate Pre-Clinical Immunogenicity and Protection Results

						Protection against‡		
CCHF antigen target	Vaccine type	No. doses; route	CCHFV IgG	CCHFV neutralizing antibodies	CCHFV T-cell response†	Symptomatic disease	Lethal disease, % survival	Ref.
Gc only	DNA fused to lysosome-associated membrane protein 1	3; IM	Yes	Yes	Yes	NA	NA	Hu et al. (*1*)
	Protein subunit anchored to gram positive enhancer matrix	3; SC	Yes	Yes	Yes	NA	NA	Wang et al. (*2*)
	Protein subunit anchored to gram positive enhancer matrix with ISA201VG and PolyI:C adjuvant	3; SC	Yes	Yes	Yes	NA	NA	Wang et al. (*2*)
	Protein subunit with Sigma adjuvant system	2; IP	Yes	Yes	NA	No	0	Kortekaas et al. (*3*)
Gn only	DNA fused to lysosome-associated membrane protein 1	3; IM	Yes	Yes	Yes	NA	NA	Hu et al. (*1*)
	Protein subunit anchored to gram positive enhancer matrix	3; SC	Yes	No	Yes	NA	NA	Wang et al. (*2*)
	Protein subunit anchored to	3; SC	Yes	No	Yes	NA	NA	Wang et al. (*2*)

CCHF antigen target	Vaccine type	No. doses; route	CCHFV IgG	CCHFV neutralizing antibodies	CCHFV T-cell response†	Protection against‡ Symptomatic disease	Protection against‡ Lethal disease, % survival	Ref.
	gram positive enhancer matrix with ISA201VG and PolyI:C adjuvant							
	Protein subunit with Sigma adjuvant system	2; IP	Yes	Yes	NA	No	NA	Kortekaas et al. (*3*)
Gc and Gn	Protein subunit produced in mosaic tobacco plants	5; oral	Yes§	NA	NA	NA	NA	Ghiasi et al. (*4*)
	Nucleoside-modified mRNA lipid nanoparticles	2; ID	Yes	Yes	Yes	Yes	100	Appelberg et al. (*5*)
	Ubiquitin-linked DNA	3; IM with electroporation	No	No	Yes	No	NA¶	Hawman et al. (*6*)
G-NAb (glycoprotein epitope spanning amino acids 1443–1566 on the M genome segment)	Protein subunit anchored to gram positive enhancer matrix	3; SC	Yes	No	Yes	NA	NA	Wang et al. (*2*)
	Protein subunit anchored to gram positive enhancer matrix with ISA201VG and PolyI:C adjuvant	3; SC	Yes	No	Yes	NA	NA	Wang et al. (*2*)
GP38 only	DNA	3; IM with electroporation	Yes	NA	NA	Partial	20	Suschak et al. (*7*)
M genome segment proteins except for GP38 and mucin-like domain	DNA	3; IM with electroporation	Yes	NA	NA	No	0	Suschak et al. (*7*)
All M genome segment proteins (all glycoprotein components and nonstructural proteins)	Alphavirus-based replicon RNA	2; IM	Possible (IgG antibodies in only 25% of vaccinated animals)	No	Yes	No	37.5	Leventhal et al. (*8*)
	DNA	3; transcutaneous via gene gun	Yes	~50%	NA	NA	NA	Spik et al. (*9*)

CCHF antigen target	Vaccine type	No. doses; route	CCHFV IgG	CCHFV neutralizing antibodies	CCHFV T-cell response†	Protection against‡ Symptomatic disease	Protection against‡ Lethal disease, % survival	Ref.
	DNA	3; IM with electroporation	Yes	Yes	NA	Partial	>60	Garrison et al. (*10*)
	DNA	3; IM with electroporation	Yes	NA	Yes	No	80–100	Suschak et al. (*7*)
	Modified vaccinia Ankara vector	2; IM	Yes	NA	Yes	Yes	100	Buttigieg et al. (*11*) Dowall et al. (*12*)
	Vesicular stomatitis virus vector (replication incompetent)	2; IP	NA	NA	NA	No	0	Rodriguez et al. (*13*)
	Vesicular stomatitis virus vector (replication competent)	1; IP	Yes	Yes	NA	No	100	Rodriguez et al. (*13*)
	Vesicular stomatitis virus vector (replication competent)	2; IP	Yes	Yes	NA	Yes	100	Rodriguez et al. (*13*)
Nucleoprotein	Alphavirus-based replicon RNA	1; IM	Yes	NA	Yes (low levels)	Partial	100	Leventhal et al. (*8*)
	Alphavirus-based replicon RNA	2; IM	Yes	No	Yes (low levels)	Yes	100	Leventhal et al. (*8*)
	Bovine herpesvirus type 4 vector	2; IP	Yes	No	Yes	No	100	Farzani et al. (*14*)
	DNA fused to lysosome-associated membrane protein 1	3; IM	Yes	Yes	Yes	NA	NA	Hu et al. (*1*)
	DNA-based Sindbis virus replicon	3; IM	Yes	NA	Yes	NA	NA	Tipih et al. (*15*)
	DNA-based Sindbis virus replicon adjuvanted with PolyI:C	3; IM	Yes	NA	Yes	NA	NA	Tipih et al. (*15*)
	DNA adjuvanted with CD24	2; IM	Yes	No	Yes	Partial	100	Farzani et al. (*16*)
	DNA	2; IM	Yes	No	Yes	Partial	100	Farzani et al. (*16*)
	DNA	2; IM	Yes	No	Yes	No	75	Farzani et al. (*14*)
	Human adenovirus 5 vector	2; IP	Yes	No	Yes	No	100	Farzani et al. (*14*)

CCHF antigen target	Vaccine type	No. doses; route	CCHFV IgG	CCHFV neutralizing antibodies	CCHFV T-cell response†	Protection against‡		Ref.
						Symptomatic disease	Lethal disease, % survival	
	Nucleoside-modified mRNA lipid nanoparticles	2; ID	Yes	No	Yes	Yes	100	Appelberg et al. (*5*)
	Modified vaccinia Ankara vector	2; IM	Yes	NA	Yes	No	0	Dowall et al. (*12*)
	Transcriptionally competent viral like particle	3; IM with electroporation	Yes	No	Yes	Yes	NA¶	Hawman et al. (*17*)
	Transcriptionally competent viral like particle	3; IP	Yes	Yes	NA	No	40	Hinkula et al. (*18*)
	Ubiquitin-linked DNA	2; IM with electroporation	Yes	No	Yes	No	NA¶	Hawman et al. (*6*)
	Ubiquitin-linked DNA	3; IM with electroporation	Yes	No	No	No	NA¶	Hawman et al. (*6*)
Nucleoprotein and all M genome segment proteins	Alphavirus-based replicon RNA	1; IM	Yes	NA	Yes	Yes	100	Leventhal et al. (*8*)
	Alphavirus-based replicon RNA	2; IM	Yes	No	Yes	Yes	100	Leventhal et al. (*8*)
Nucleoprotein, Gc, and Gn	Nucleoside-modified mRNA lipid nanoparticles	2; ID	Yes	Yes	Yes	Yes	100	Appelberg et al. (*5*)
	Ubiquitin-linked DNA	3; ID with electroporation	Yes	Yes	NA	Yes	100	Hinkula et al. (*18*)
All CCHF viral proteins	Viral replicon particle containing L and S genome segments#	1; SC	Yes	NA	NA	Yes	100	Scholte et al. (*19*)
			Yes	NA	NA	Yes	100	Spengler et al. (*20*)
			NA	NA	NA	Vaccinated ≥7 d before viral challenge: Yes. Vaccinated 3 d before viral challenge: Partial. Vaccinated 1 d before or after viral challenge: No	Vaccinated ≥3 d before viral challenge,100. Vaccinated 1 d before or after viral challenge, 0	Spengler et al. (*21*)
	Inactivated whole virus	3; IP	Yes	Yes	Yes	Partial	80–100	Berber et al. (*22*)

CCHF antigen target	Vaccine type	No. doses; route	CCHFV IgG	CCHFV neutralizing antibodies	CCHFV T-cell response†	Protection against‡		Ref.
						Symptomatic disease	Lethal disease, % survival	
	adjuvanted with alum derived from cell culture							Canakoglu et al. (*23*) Pavel et al. (*24*)

*CCHFV, Crimean-Congo hemorrhagic fever virus; ID, intradermal; IM, intramuscular; IP, intraperitoneal; SC, subcutaneous; NA, Not assessed.
†Direct measurement of CCHFV, CCHFV antigen-stimulated T-cell, or splenocyte responses only. Studies measuring serum cytokine levels post-vaccination were excluded.
‡For studies that assessed vaccine dosing ranges, the protection against symptomatic disease or lethal disease is presented only for the highest dose used.
§IgA antibodies were also detectable in serum and fecal pellets from vaccinated mice.
¶Unable to assess survival benefit due to non-lethal disease in the unvaccinated animal comparator study arm.
#The M genome segment is not contained in the viral replicon particle, however proteins from the M genome segment are provided *in trans* during the creation of the viral replicon particle leading to the incorporation of M genome segment proteins on the surface of the viral replicon particle.

References

1. Hu YL, Zhang LQ, Liu XQ, Ye W, Zhao YX, Zhang L, et al. Construction and evaluation of DNA vaccine encoding Crimean Congo hemorrhagic fever virus nucleocapsid protein, glycoprotein N-terminal and C-terminal fused with LAMP1. Front Cell Infect Microbiol. 2023;13:1121163. PubMed https://doi.org/10.3389/fcimb.2023.1121163

2. Wang Q, Wang S, Shi Z, Li Z, Zhao Y, Feng N, et al. GEM-PA–based subunit vaccines of Crimean Congo hemorrhagic fever induces systemic immune responses in mice. Viruses. 2022;14:1664. PubMed https://doi.org/10.3390/v14081664

3. Kortekaas J, Vloet RP, McAuley AJ, Shen X, Bosch BJ, de Vries L, et al. Crimean-Congo Hemorrhagic fever virus subunit vaccines induce high levels of neutralizing antibodies but no protection in STAT1 knockout mice. Vector Borne Zoonotic Dis. 2015;15:759–64. PubMed https://doi.org/10.1089/vbz.2015.1855

4. Ghiasi SM, Salmanian AH, Chinikar S, Zakeri S. Mice orally immunized with a transgenic plant expressing the glycoprotein of Crimean-Congo hemorrhagic fever virus. Clin Vaccine Immunol. 2011;18:2031–7. PubMed https://doi.org/10.1128/CVI.05352-11

5. Appelberg S, John L, Pardi N, Végvári Á, Bereczky S, Ahlén G, et al. Nucleoside-modified mRNA vaccines protect IFNAR$^{-/-}$ mice against Crimean-Congo hemorrhagic fever virus infection. J Virol. 2022;96:e0156821. PubMed https://doi.org/10.1128/jvi.01568-21

6. Hawman DW, Meade-White K, Leventhal S, Appelberg S, Ahlén G, Nikouyan N, et al. Accelerated DNA vaccine regimen provides protection against Crimean-Congo hemorrhagic fever virus challenge in a macaque model. Mol Ther. 2023;31:387–97. PubMed https://doi.org/10.1016/j.ymthe.2022.09.016

7. Suschak JJ, Golden JW, Fitzpatrick CJ, Shoemaker CJ, Badger CV, Schmaljohn CS, et al. A CCHFV DNA vaccine protects against heterologous challenge and establishes GP38 as immunorelevant in mice. NPJ Vaccines. 2021;6:31. PubMed https://doi.org/10.1038/s41541-021-00293-9

8. Leventhal SS, Meade-White K, Rao D, Haddock E, Leung J, Scott D, et al. Replicating RNA vaccination elicits an unexpected immune response that efficiently protects mice against lethal Crimean-Congo hemorrhagic fever virus challenge. EBioMedicine. 2022;82:104188. PubMed https://doi.org/10.1016/j.ebiom.2022.104188

9. Spik K, Shurtleff A, McElroy AK, Guttieri MC, Hooper JW, SchmalJohn C. Immunogenicity of combination DNA vaccines for Rift Valley fever virus, tick-borne encephalitis virus, Hantaan virus, and Crimean Congo hemorrhagic fever virus. Vaccine. 2006;24:4657–66. PubMed https://doi.org/10.1016/j.vaccine.2005.08.034

10. Garrison AR, Shoemaker CJ, Golden JW, Fitzpatrick CJ, Suschak JJ, Richards MJ, et al. A DNA vaccine for Crimean-Congo hemorrhagic fever protects against disease and death in two lethal mouse models. PLoS Negl Trop Dis. 2017;11:e0005908. PubMed https://doi.org/10.1371/journal.pntd.0005908

11. Buttigieg KR, Dowall SD, Findlay-Wilson S, Miloszewska A, Rayner E, Hewson R, et al. A novel vaccine against Crimean-Congo haemorrhagic fever protects 100% of animals against lethal challenge in a mouse model. PLoS One. 2014;9:e91516. PubMed https://doi.org/10.1371/journal.pone.0091516

12. Dowall SD, Graham VA, Rayner E, Hunter L, Watson R, Taylor I, et al. Protective effects of a modified vaccinia Ankara-based vaccine candidate against Crimean-Congo haemorrhagic fever virus require both cellular and humoral responses. PLoS One. 2016;11:e0156637. PubMed https://doi.org/10.1371/journal.pone.0156637

13. Rodriguez SE, Cross RW, Fenton KA, Bente DA, Mire CE, Geisbert TW. Vesicular stomatitis virus-based vaccine protects mice against Crimean-Congo hemorrhagic fever. Sci Rep. 2019;9:7755. PubMed https://doi.org/10.1038/s41598-019-44210-6

14. Aligholipour Farzani T, Földes K, Hanifehnezhad A, Yener Ilce B, Bilge Dagalp S, Amirzadeh Khiabani N, et al. Bovine herpesvirus type 4 (BoHV-4) vector delivering nucleocapsid protein of Crimean-Congo hemorrhagic fever virus induces comparable protective immunity against lethal challenge in IFNα/β/γR−/− mice models. Viruses. 2019;11:237. PubMed https://doi.org/10.3390/v11030237

15. Tipih T, Heise M, Burt FJ. Immunogenicity of a DNA-Based Sindbis Replicon Expressing Crimean-Congo Hemorrhagic Fever Virus Nucleoprotein. Vaccines (Basel). 2021;9:1491. PubMed https://doi.org/10.3390/vaccines9121491

16. Aligholipour Farzani T, Hanifehnezhad A, Földes K, Ergünay K, Yilmaz E, Hashim Mohamed Ali H, et al. Co-delivery effect of CD24 on the immunogenicity and lethal challenge protection of a DNA vector expressing nucleocapsid protein of Crimean Congo hemorrhagic fever virus. Viruses. 2019;11:75. PubMed https://doi.org/10.3390/v11010075

17. Hawman DW, Ahlén G, Appelberg KS, Meade-White K, Hanley PW, Scott D, et al. A DNA-based vaccine protects against Crimean-Congo haemorrhagic fever virus disease in a cynomolgus macaque model. Nat Microbiol. 2020;6:187–95. PubMed https://doi.org/10.1038/s41564-020-00815-6

18. Hinkula J, Devignot S, Åkerström S, Karlberg H, Wattrang E, Bereczky S, et al. Immunization with DNA plasmids coding for Crimean-Congo hemorrhagic fever virus capsid and envelope proteins and/or virus-like particles induces protection and survival in challenged mice. J Virol. 2017;91:e02076-16. PubMed https://doi.org/10.1128/JVI.02076-16

19. Scholte FEM, Spengler JR, Welch SR, Harmon JR, Coleman-McCray JD, Freitas BT, et al. Single-dose replicon particle vaccine provides complete protection against Crimean-Congo hemorrhagic fever virus in mice. Emerg Microbes Infect. 2019;8:575–8. PubMed https://doi.org/10.1080/22221751.2019.1601030

20. Spengler JR, Welch SR, Scholte FEM, Coleman-McCray JD, Harmon JR, Nichol ST, et al. Heterologous protection against Crimean-Congo hemorrhagic fever in mice after a single dose of replicon particle vaccine. Antiviral Res. 2019;170:104573. PubMed https://doi.org/10.1016/j.antiviral.2019.104573

21. Spengler JR, Welch SR, Scholte FEM, Rodriguez SE, Harmon JR, Coleman-McCray JD, et al. Viral replicon particles protect IFNAR$^{-/-}$ mice against lethal Crimean-Congo hemorrhagic fever virus challenge three days after vaccination. Antiviral Res. 2021;191:105090. PubMed https://doi.org/10.1016/j.antiviral.2021.105090

22. Berber E, Çanakoğlu N, Tonbak Ş, Ozdarendeli A. Development of a protective inactivated vaccine against Crimean-Congo hemorrhagic fever infection. Heliyon. 2021;7:e08161. PubMed https://doi.org/10.1016/j.heliyon.2021.e08161

23. Canakoglu N, Berber E, Tonbak S, Ertek M, Sozdutmaz I, Aktas M, et al. Immunization of knock-out α/β interferon receptor mice against high lethal dose of Crimean-Congo hemorrhagic fever virus with a cell culture based vaccine. PLoS Negl Trop Dis. 2015;9:e0003579. PubMed https://doi.org/10.1371/journal.pntd.0003579

24. Pavel STI, Yetiskin H, Kalkan A, Ozdarendeli A. Evaluation of the cell culture based and the mouse brain derived inactivated vaccines against Crimean-Congo hemorrhagic fever virus in transiently immune-suppressed (IS) mouse model. PLoS Negl Trop Dis. 2020;14:e0008834. PubMed https://doi.org/10.1371/journal.pntd.0008834
