# Supplementary material for: Crimean-Congo Hemorrhagic Fever Virus for Clinicians—Diagnosis, Clinical Management, and Therapeutics
Source: Emerg Infect Dis. 2024 May;30(5):864–73. doi: 10.3201/eid3005.231648 (PMC11060459; doi:10.3201/eid3005.231648)
Supplement: Appendix 2 — Additional references for clinicians on Crimean-Congo hemorrhagic fever virus diagnosis, clinical management, and therapeutics. [file 23-1648-Techapp-s2.pdf]

Article DOI: https://doi.org/10.3201/eid3005.231648

EID cannot ensure accessibility for supplementary materials supplied by authors. Readers who have difficulty accessing supplementary content should contact the authors for assistance.

Crimean-Congo Hemorrhagic Fever Virus for Clinicians—Diagnosis, Clinical Management, and Therapeutics

Appendix 2

Additional References

51. Watts DM, Ussery MA, Nash D, Peters CJ. Inhibition of Crimean-Congo hemorrhagic fever viral infectivity yields in vitro by ribavirin. Am J Trop Med Hyg. 1989;41:581–5. PubMed https://doi.org/10.4269/ajtmh.1989.41.581

52. Paragas J, Whitehouse CA, Endy TP, Bray M. A simple assay for determining antiviral activity against Crimean-Congo hemorrhagic fever virus. Antiviral Res. 2004;62:21–5. PubMed https://doi.org/10.1016/j.antiviral.2003.11.006

53. Celikbas AK, Dokuzoğuz B, Baykam N, Gok SE, Eroğlu MN, Midilli K, et al. Crimean-Congo hemorrhagic fever among health care workers, Turkey. Emerg Infect Dis. 2014;20:477–9. PubMed https://doi.org/10.3201/eid2003.131353

54. Conger NG, Paolino KM, Osborn EC, Rusnak JM, Günther S, Pool J, et al. Health care response to CCHF in US soldier and nosocomial transmission to health care providers, Germany, 2009. Emerg Infect Dis. 2015;21:23–31. PubMed https://doi.org/10.3201/eid2101.141413

55. Guven G, Talan L, Altintas ND, Memikoglu KO, Yoruk F, Azap A. An unexpected fatal CCHF case and management of exposed health care workers. Int J Infect Dis. 2017;55:118–21. PubMed https://doi.org/10.1016/j.ijid.2016.12.026

56. Tütüncü EE, Gurbuz Y, Ozturk B, Kuscu F, Sencan I. Crimean Congo haemorrhagic fever, precautions and ribavirin prophylaxis: a case report. Scand J Infect Dis. 2009;41:378–80. PubMed https://doi.org/10.1080/00365540902882434

57. Saleem J, Usman M, Nadeem A, Sethi SA, Salman M. Crimean-Congo hemorrhagic fever: a first case from Abbottabad, Pakistan. Int J Infect Dis. 2009;13:e121–3. PubMed https://doi.org/10.1016/j.ijid.2008.07.023

58. Bangash SA, Khan EA. Treatment and prophylaxis with ribavirin for Crimean-Congo hemorrhagic fever—is it effective? J Pak Med Assoc. 2003;53:39–41. PubMed

59. Roberts SS. Assessing ribavirin exposure during pregnancy: the Ribavirin Pregnancy Registry. Gastroenterol Nurs. 2008;31:413–7. PubMed https://doi.org/10.1097/SGA.0b013e31818eb70d

60. Hawman DW, Haddock E, Meade-White K, Williamson B, Hanley PW, Rosenke K, et al. Favipiravir (T-705) but not ribavirin is effective against two distinct strains of Crimean-Congo hemorrhagic fever virus in mice. Antiviral Res. 2018;157:18–26. PubMed https://doi.org/10.1016/j.antiviral.2018.06.013

61. Tignor GH, Hanham CA. Ribavirin efficacy in an in vivo model of Crimean-Congo hemorrhagic fever virus (CCHF) infection. Antiviral Res. 1993;22:309–25. PubMed https://doi.org/10.1016/0166-3542(93)90040-P

62. Bodur H, Erbay A, Akıncı E, Öngürü P, Bayazıt N, Eren SS, et al. Effect of oral ribavirin treatment on the viral load and disease progression in Crimean-Congo hemorrhagic fever. Int J Infect Dis. 2011;15:e44–7. PubMed https://doi.org/10.1016/j.ijid.2010.09.009

63. D'Addiego J, Elaldi N, Wand N, Osman K, Bagci BK, Kennedy E, et al. Investigating the effect of ribavirin treatment on genetic mutations in Crimean-Congo haemorrhagic fever virus (CCHFV) through next-generation sequencing. J Med Virol. 2023;95:e28548. PubMed https://doi.org/10.1002/jmv.28548

64. Ascioglu S, Leblebicioglu H, Vahaboglu H, Chan KA. Ribavirin for patients with Crimean-Congo haemorrhagic fever: a systematic review and meta-analysis. J Antimicrob Chemother. 2011;66:1215–22. PubMed https://doi.org/10.1093/jac/dkr136

65. Arab-Bafrani Z, Jabbari A, Mostakhdem Hashemi M, Arabzadeh AM, Gilanipour A, Mousavi E. Identification of the crucial parameters regarding the efficacy of ribavirin therapy in Crimean-Congo haemorrhagic fever (CCHF) patients: a systematic review and meta-analysis. J Antimicrob Chemother. 2019;74:3432–9. PubMed https://doi.org/10.1093/jac/dkz328

66. Johnson S, Henschke N, Maayan N, Mills I, Buckley BS, Kakourou A, et al. Ribavirin for treating Crimean Congo haemorrhagic fever. Cochrane Database Syst Rev. 2018;6:CD012713. PubMed https://doi.org/10.1002/14651858.CD012713.pub2

67. Soares-Weiser K, Thomas S, Thomson G, Garner P. Ribavirin for Crimean-Congo hemorrhagic fever: systematic review and meta-analysis. BMC Infect Dis. 2010;10:207. PubMed https://doi.org/10.1186/1471-2334-10-207

68. Koksal I, Yilmaz G, Aksoy F, Aydin H, Yavuz I, Iskender S, et al. The efficacy of ribavirin in the treatment of Crimean-Congo hemorrhagic fever in eastern Black Sea region in Turkey. J Clin Virol. 2010;47:65–8. PubMed https://doi.org/10.1016/j.jcv.2009.11.007

69. Tchesnokov EP, Bailey-Elkin BA, Mark BL, Götte M. Independent inhibition of the polymerase and deubiquitinase activities of the Crimean-Congo hemorrhagic fever virus full-length L-protein. PLoS Negl Trop Dis. 2020;14:e0008283. PubMed https://doi.org/10.1371/journal.pntd.0008283

70. Oestereich L, Rieger T, Neumann M, Bernreuther C, Lehmann M, Krasemann S, et al. Evaluation of antiviral efficacy of ribavirin, arbidol, and T-705 (favipiravir) in a mouse model for Crimean-Congo hemorrhagic fever. PLoS Negl Trop Dis. 2014;8:e2804. PubMed https://doi.org/10.1371/journal.pntd.0002804

71. Hawman DW, Haddock E, Meade-White K, Nardone G, Feldmann F, Hanley PW, et al. Efficacy of favipiravir (T-705) against Crimean-Congo hemorrhagic fever virus infection in cynomolgus macaques. Antiviral Res. 2020;181:104858. PubMed https://doi.org/10.1016/j.antiviral.2020.104858

72. Dülger AC, Yakarişik M, Uzun YE, Şahin AM. Treatment of Crimean-Congo haemorrhagic fever by favipiravir in a patient with novel coronavirus co-infection. Eur J Case Rep Intern Med. 2020;7:002042. PubMed

73. Pilkington V, Pepperrell T, Hill A. A review of the safety of favipiravir—a potential treatment in the COVID-19 pandemic? J Virus Erad. 2020;6:45–51. PubMed https://doi.org/10.1016/S2055-6640(20)30016-9

74. Ohlstein EH, Vickery L, Sauermelch C, Willette RN. Vasodilation induced by endothelin: role of EDRF and prostanoids in rat hindquarters. Am J Physiol. 1990;259:H1835–41. PubMed

75. Ergonul O. Treatment of Crimean-Congo hemorrhagic fever. Antiviral Res. 2008;78:125–31. PubMed https://doi.org/10.1016/j.antiviral.2007.11.002

76. Fillâtre P, Revest M, Tattevin P. Crimean-Congo hemorrhagic fever: an update. Med Mal Infect. 2019;49:574–85. PubMed https://doi.org/10.1016/j.medmal.2019.09.005

77. Mardani M, Rahnavardi M, Sharifi-Mood B. Current treatment of Crimean-Congo hemorrhagic fever in children. Expert Rev Anti Infect Ther. 2010;8:911–8. PubMed https://doi.org/10.1586/eri.10.67

78. Berber I, Korkmaz S, Sarici A, Erkurt MA, Kuku I, Kaya E, et al. Therapeutic plasma exchange for envenomation: Is it reasonable? Transfus Apheresis Sci. 2021;60:103241. PubMed https://doi.org/10.1016/j.transci.2021.103241

79. Beştepe Dursun Z, Korkmaz S, Türe Z, Kaynar L, Dursun A, Çelik İ. Efficacy of therapeutic plasma exchange in patients with Crimean-Congo hemorrhagic fever. J Clin Apher. 2021;36:390–7. PubMed https://doi.org/10.1002/jca.21875

80. Meço BC, Memikoğlu O, Ilhan O, Ayyıldız E, Gunt C, Unal N, et al. Double filtration plasmapheresis for a case of Crimean-Congo hemorrhagic fever. Transfus Apheresis Sci. 2013;48:331–4. PubMed https://doi.org/10.1016/j.transci.2013.04.011

81. Perk O, Emeksiz S, Ozcan S, Meral G. Crimean-Congo hemorrhagic fever: A pediatric case responding to plasmapheresis treatment. Transfus Apheresis Sci. 2021;60:103215. PubMed https://doi.org/10.1016/j.transci.2021.103215

82. Oygar PD, Gürlevik SL, Sağ E, İlbay S, Aksu T, Demir OO, et al. Changing disease course of Crimean-Congo hemorrhagic fever in children, Turkey. Emerg Infect Dis. 2023;29:268–77. PubMed https://doi.org/10.3201/eid2902.220976

83. Dilber E, Cakir M, Erduran E, Koksal I, Bahat E, Mutlu M, et al. High-dose methylprednisolone in children with Crimean-Congo haemorrhagic fever. Trop Doct. 2010;40:27–30. PubMed https://doi.org/10.1258/td.2009.090069

84. Sharifi-Mood B, Alavi-Naini R, Metanat M, Mohammadi M, Shakeri A, Amjadi A. Efficacy of high-dose methylprednisolone in patients with Crimean-Congo haemorrhagic fever and severe thrombocytopenia. Trop Doct. 2013;43:49–53. PubMed https://doi.org/10.1177/0049475513486642

85. Dokuzoguz B, Celikbas AK, Gök ŞE, Baykam N, Eroglu MN, Ergönül Ö. Severity scoring index for Crimean-Congo hemorrhagic fever and the impact of ribavirin and corticosteroids on fatality. Clin Infect Dis. 2013;57:1270–4. PubMed https://doi.org/10.1093/cid/cit527

86. Vassilenko SM, Vassilev TL, Bozadjiev LG, Bineva IL, Kazarov GZ. Specific intravenous immunoglobulin for Crimean-Congo haemorrhagic fever. Lancet. 1990;335:791–2. PubMed https://doi.org/10.1016/0140-6736(90)90906-L

87. van Eeden PJ, van Eeden SF, Joubert JR, King JB, van de Wal BW, Michell WL. A nosocomial outbreak of Crimean-Congo haemorrhagic fever at Tygerberg Hospital. Part II. Management of patients. S Afr Med J. 1985;68:718–21. PubMed

88. Kubar A, Haciomeroglu M, Ozkul A, Bagriacik U, Akinci E, Sener K, et al. Prompt administration of Crimean-Congo hemorrhagic fever (CCHF) virus hyperimmunoglobulin in patients diagnosed with CCHF and viral load monitorization by reverse transcriptase-PCR. Jpn J Infect Dis. 2011;64:439–43. PubMed https://doi.org/10.7883/yoken.64.439

89. Golden JW, Shoemaker CJ, Lindquist ME, Zeng X, Daye SP, Williams JA, et al. GP38-targeting monoclonal antibodies protect adult mice against lethal Crimean-Congo hemorrhagic fever virus infection. Sci Adv. 2019;5:eaaw9535. PubMed https://doi.org/10.1126/sciadv.aaw9535

90. Fels JM, Maurer DP, Herbert AS, Wirchnianski AS, Vergnolle O, Cross RW, et al. Protective neutralizing antibodies from human survivors of Crimean-Congo hemorrhagic fever. Cell. 2021;184:3486–3501.e21. PubMed https://doi.org/10.1016/j.cell.2021.05.001
